# 含伊沙佐米方案治疗复发/难治多发性骨髓瘤患者的疗效和生存分析

**DOI:** 10.3760/cma.j.issn.0253-2727.2022.08.011

**Published:** 2022-08

**Authors:** 辰 杨, 俊玲 庄

**Affiliations:** 中国医学科学院、北京协和医学院北京协和医院血液内科，北京 100730 Department of Hematology, Peking Union Medical College Hospital, Chinese Academy of Medical Science and Peking Union Medical College, Beijing 100730, China

近20年来，随着蛋白酶体抑制剂（PI）硼替佐米及免疫调节剂（IMiD）来那度胺等药物的广泛使用，多发性骨髓瘤（MM）患者的疗效和生存显著改善，再加上持续治疗的整体管理模式，MM逐渐演变成一种可以控制的慢性肿瘤[1]。由于MM仍不可治愈，长期用药的方便性和安全性仍是治疗需要考虑的主要问题之一[2]。

伊沙佐米是二代口服蛋白酶体抑制剂，2018年正式在中国上市。上市前的Ⅲ期临床研究TOURMALINE-MM1及其在中国的延展试验显示，伊沙佐米+来那度胺+地塞米松（IRd方案）治疗复发/难治MM（RRMM）有较好的疗效和安全性，明显延长了患者的无进展生存（PFS）时间，与对照组比较，大大降低了患者的死亡风险，且周围神经病（PN）发生率低[3]–[4]。由于口服药物方便，真实世界研究（RWS）显示伊沙佐米应用的疗程与临床试验（RCT）接近，显示出更为实用的优势[5]。由于药物进入中国时间不长，伊沙佐米用于真实世界RRMM患者的临床报道有限[6]。为此，我们回顾性分析了2018年7月1日至2021年10月31日于北京协和医院接受至少2个周期伊沙佐米治疗的RRMM患者，进一步探索伊沙佐米在临床应用中的疗效、安全性，分析适合治疗的患者人群及时机。

## 病例与方法

1. 病例及临床资料：纳入2018年7月1日至2021年10月31日于北京协和医院至少完成2个疗程含伊沙佐米方案治疗的73例RRMM患者，复发/难治的定义采用国际骨髓瘤工作组（IMWG）标准。基线数据和疗效评估数据相对完整，根据国际分期系统（ISS）和国际分期系统修订版（R-ISS）[2]进行临床分期。依据R-ISS，细胞遗传学高危定义为骨髓CD138分选后行荧光原位杂交（FISH）检测出17号染色体缺失［del（17p）］、免疫球蛋白重链（IGH）染色体易位［t（4;14）、t（14;16）］。根据RRMM患者接受的治疗方案分为IRd组和其他方案组。

2. 治疗方案：根据《中国多发性骨髓瘤诊治指南（2022年修订）》[2]，治疗方案如下：①IRd方案，具体为：伊沙佐米3 mg/d或4 mg/d，口服，第1、8、15天；来那度胺25 mg/d，隔日口服，第1～21天；地塞米松20～40 mg/d，口服，第1、8、15、22天，每28 d为1个疗程。②ITd方案：沙利度胺100 mg/d，晚间口服；伊沙佐米、地塞米松剂量同IRd方案。③ICd方案：环磷酰胺300 mg·m^−2^·d^−1^，口服，第1、8、15天；伊沙佐米、地塞米松剂量同IRd方案。④IPd方案：泊马度胺4 mg/d，口服，第1～21天；伊沙佐米、地塞米松剂量同IRd方案。⑤IAd方案：脂质体阿霉素20～25 mg·m^−2^·d^−1^，第1天，静脉滴注；伊沙佐米、地塞米松剂量同IRd方案。⑥IMd方案：美法仑2 mg/d，第1～4天；伊沙佐米、地塞米松剂量同IRd方案。⑦Dara+Id：达雷妥尤单抗16 mg/kg，按照推荐方式给药；伊沙佐米、地塞米松剂量同IRd方案。以上方案中，年龄>75岁、美国东部肿瘤协作组（ECOG）评分≥2分患者地塞米松剂量减半。

3. 疗效评估：每周期治疗结束后依据IMWG标准进行疗效评估[7]。总反应率（ORR）为达到部分缓解（PR）及以上患者所占比例。临床复发、侵袭性复发、生化复发的定义参见《中国多发性骨髓瘤诊治指南（2022年修订）》[2]。

4. 随访：所有病例随访至2021年10月31日，通过北京协和医院电子病历系统及电话进行随访。对于随访期间死亡的病例，根据病历记录或电话联系确认。总生存（OS）时间为自首次应用伊沙佐米至患者死亡或随访终止时间。PFS时间为自首次应用伊沙佐米至疾病进展、患者死亡或随访终止时间，PFS_1_时间为自开始治疗至首次复发的时间。失访患者2例（2.90％），中位随访时间24（2～44）个月。

5. 统计学处理：采用SPSS 26.0软件进行统计学分析。计数资料用例数（百分比）表示，计量资料用中位数（范围）表示。采用Kaplan-Meier法绘制生存曲线，Log-rank检验进行单因素分析，分类变量的组间比较采用*χ*^2^检验，当不满足*χ*^2^检验时采用Fisher检验，采用Cox比例风险模型进行多因素分析。单侧检验水准*α*＝0.05，双侧*P*<0.05为差异有统计学意义。

## 结果

1. 患者的临床特征：共纳入73例RRMM患者，基线资料见表1。中位年龄为64.5（43～90）岁，男40例（54.8％）。>75岁患者25例（34.2％），37例（50.7％）年龄<65岁。ISS Ⅲ期患者41例（56.2％），R-ISS Ⅲ期患者22例（30％），高危细胞遗传学异常患者11例（25％，11/44），1q21+患者21例（28.8％）。自体造血干细胞移植（ASCT）后复发患者8例（11％），继发浆细胞白血病患者3例，合并骨旁或髓外浆细胞瘤患者12例，19例合并肾功能不全患者中9例接受透析治疗。56例（76.7％）患者合并冠心病、高血压、2型糖尿病、肾病、脑血管病、心律失常、哮喘、支气管扩张等。既往治疗含硼替佐米方案患者61例（83.6％）、含来那度胺方案患者49例（67.1％）。将RRMM患者分为IRd组和其他方案组（表1）。

**表1:** 73例复发/难治多发性骨髓瘤患者的临床特征

特征	总体（73例）	IRd组（40例）	其他方案组（33例）
年龄［岁，*M*（范围）］	64（43～90）	63（43～90）	69（49～83）
<65岁［例（％）］	37（50.7）	24（60.0）	13（39.4）
65～74岁［例（％）］	11（15.1）	8（20.0）	9（27.3）
≥75岁［例（％）］	25（34.2）	8（20.0）	11（33.3）
男性［例（％）］	40（54.8）	21（52.5）	20（60.6）
M蛋白类型（例，IgG/IgA/轻链/IgD）	30/20/16/8	20/7/8/5	10/12/8/3
ECOG评分≥2分［例（％）］	36（49.3）	13（32.5）	15（45.5）
ISS分期［例（％）］			
Ⅰ期	13（17.8）	8（20.0）	5（15.2）
Ⅱ期	19（26.0）	9（22.5）	10（30.3）
Ⅲ期	41（56.2）	23（57.5）	18（54.5）
高危细胞遗传学异常［阳性例数/总例数］	11/44	10/24	1/20
血肌酐>177 µmol/L［例（％）］	19（26.0）	7（17.5）	12（36.4）
髓外病变［例（％）］	12（16.4）	8（20.0）	4（12.1）
治疗线数［例（％）］			
2线	46（63.0）	25（62.5）	21（63.6）
3线	22（30.1）	12（30.0）	10（30.3）
≥4线	5（6.8）	3（7.5）	2（6.1）
前线治疗［例（％）］			
硼替佐米［例（％），治疗/耐药］	61（83.6）/16（21.9）	34（85）/11（27.5）	27（81.8）/5（15.2）
来那度胺［例（％），治疗/耐药］	49（67.1）/40（54.8）	25（62.5）/17（42.5）	24（72.7）/23（69.7）
ASCT	8（11.0）	8（20.0）	0（0）
PFS_1_>24个月［例（％）］	30（41.1）	17（42.5）	13（39.4）
临床复发	48（65.8）	20（50.0）	28（84.9）
伊沙佐米应用<6个月	40（54.8）	21（52.5）	19（57.6）

注：IRd：伊沙佐米+来那度胺+地塞米松；ECOG：美国东部肿瘤协作组；ASCT：自体造血干细胞移植；PFS_1_：自开始治疗至首次复发的时间

2. 疗效和生存分析：在73例RRMM患者中，应用IRd、Id、ITd、IPd、ICd、IMd方案的患者分别为40、27、18、7、4、1例，应用Dara+Id和IAd方案的患者各3例，有9例患者应用IRd方案进展后改用伊沙佐米联合其他药物再次获得治疗反应。既往中位治疗线数为2（2～5）线，截至随访期结束，34例（46.6％）患者仍继续治疗，因疾病进展（PD）、不良反应（AE）终止治疗的患者分别为26例（35.6％）、7例（9.6％）。治疗中位疗程数为7（2～38）个，ORR为53.4％，其中完全缓解（CR）/严格意义上的完全缓解（sCR）率为4.1％，非常好的部分缓解（VGPR）率为6.8％，PR率为41.1％，中位获得反应时间为1.5个月，中位PFS时间18个月，中位OS时间未达到（图1）。细胞遗传学（CA）高危患者的中位PFS、OS时间分别为5个月、13个月，标危患者中位PFS、OS时间分别为29个月、未达到。2线、3线、≥4线应用含伊沙佐米方案的患者分别为46、22、5例，ORR分别为54.3％、51.7％、40.0％，伊沙佐米中位应用疗程分别为13、18、3个，中位PFS时间分别为13、19、4个月（图2），ISS分期Ⅲ期患者41例，其中2线、3线、≥4线应用含伊沙佐米治疗的患者分别为28例、10例、3例，CA高危的11例患者中8例应用二线治疗，3例应用三线治疗。

**图1:**
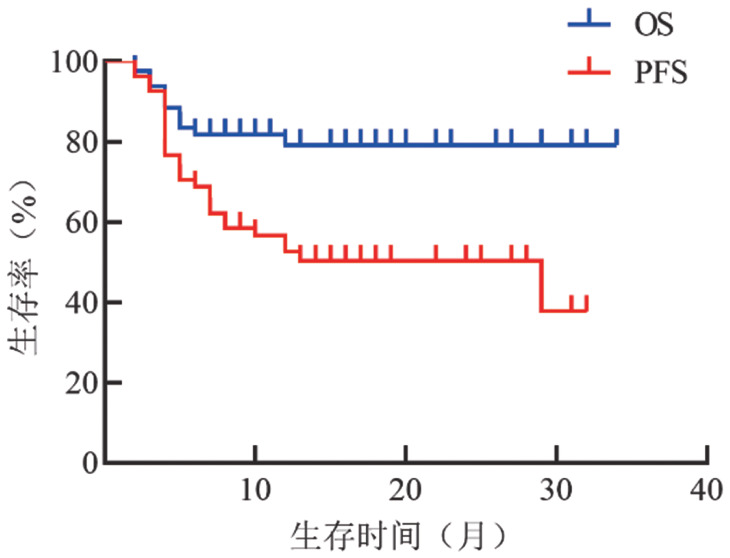
73例复发/难治多发性骨髓瘤患者的总生存（OS）和无进展生存（PFS）曲线

**图2:**
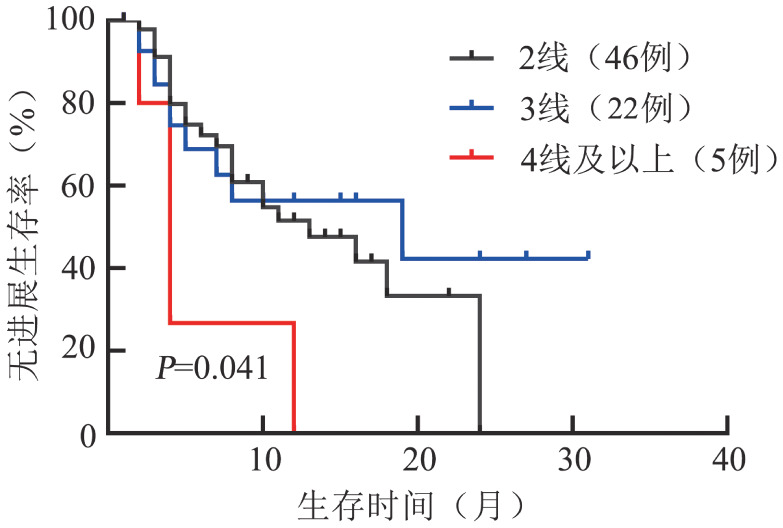
复发/难治多发性骨髓瘤患者不同线数治疗应用含伊沙佐米方案的无进展生存曲线

IRd组患者共40例，中位治疗疗程数为8（2～26）个，中位随访11（2～30）个月，ORR为40％，其中获得CR、VGPR患者各1例，14例患者获得PR；中位PFS时间8个月，中位OS时间未达到。应用IRd方案的2线、3线、≥4线患者分别为25例、12例、3例，ORR分别为36.0％、50.0％、33.3％。中位PFS时间分别为8、14、3个月，中位OS时间分别为未达到、未达到、12个月。IRd方案组中，ISS Ⅲ期患者共23例，其中2线、3线、≥4线应用含伊沙佐米方案治疗的患者分别为16例、6例、1例，CA高危的10例患者中7例应用二线治疗，3例应用三线治疗。在不同线数治疗中应用IRd方案的患者中位PFS时间的差异无统计学意义（*P*＝0.923）。高危与标危组中位PFS时间分别为4个月和10个月（95％*CI* 0.752～6.948，*P*＝0.040）。三线持续治疗至少6个疗程的患者中位PFS时间达28个月。

除IRd方案外，其他含伊沙佐米方案用于2线、3线、≥ 4线治疗的ORR分别为66.7％、52.9％、50.0％。

既往对硼替佐米耐药、来那度胺耐药或进行过ASCT后复发的RRMM患者，含伊沙佐米的方案ORR分别为55.7％、46.9％、50％，IRd方案的ORR分别为50％、44％、50％，其他方案的ORR分别为66.7％、62.5％、0。

3. 影响伊沙佐米方案疗效的相关因素：初诊时CA高危、侵袭性复发、硼替佐米耐药、治疗<6个月均是RRMM进展的独立危险因素（*P*值均<0.05），但年龄、性别、ISS分期、ECOG评分、肾功能不全、是否伴并发症、一线治疗的PFS、硼替佐米或来那度胺暴露/耐药、1q21+均不是独立危险因素（*P*值均>0.05）；多因素分析表明，CA高危、侵袭性复发影响RRMM患者的PFS（表2）。

在IRd治疗组，CA高危、侵袭性复发、IRd治疗<6个月、前线治疗硼替佐米/来那度胺耐药均为PFS的独立危险因素（*P*值均<0.05）。多因素分析表明，IRd治疗<6个月，前线治疗时来那度胺耐药影响患者的PFS（表2）。

**表2:** 影响总体及IRd方案组复发/难治多发性骨髓瘤患者无进展生存的单因素及多因素分析

因素	总体					IRd方案组				
	单因素分析		多因素分析			单因素分析		多因素分析		
	*χ*^2^值	*P*值	*OR*	95%*CI*	*P*值	*χ*^2^值	*P*值	*OR*	95%*CI*	*P*值
CA高危	10.037	0.002	3.127	1.265～7.729	0.014	8.232	0.004	3.675	1.347～10.028	0.528
侵袭性复发	6.062	0.014	2.259	1.028～4.964	0.042	8.193	0.004	6.399	1.425～28.727	0.141
Ixa治疗<6个月	6.792	0.013	93.713	11.276～78.826	<0.001	31.734	<0.001	46.833	5.772～38.008	<0.001
Len耐药	7.432	0.014	1.652	0.793～3.440	−	8.191	0.004	3.622	1.350～9.718	0.003
Bort耐药	10.745	0.009	0.959	0.443～2.076	−	11.561	0.001	4.456	1.659～11.965	0.054

注：IRd：伊沙佐米+来那度胺+地塞米松；CA：细胞遗传学；Ixa：伊沙佐米；Len：来那度胺；Bort：硼替佐米；−：无数据

4. 不良反应：伊沙佐米治疗的主要不良反应为腹泻、PN、血小板降低、乏力、感染、带状疱疹，发生率分别为39.1％、37.7％、14.5％、11.6％、7.2％、5.8％，其中3～4级不良事件总发生率为19.2％，8例发生于IRd方案治疗后，主要为腹泻、血小板减低、中性粒细胞缺乏、感染。所有患者未出现3～4级PN，且较前线治疗无加重。76％患者生活质量明显改善。3例患者因腹泻仅使用2周期后中断伊沙佐米治疗，6例患者治疗期间死亡，其中感染性休克3例，急性左心衰、急性冠脉综合征、大面积脑梗死各1例，均发生于MM进展期，且有基础合并症。

## 讨论

近年来，随着硼替佐米和来那度胺在中国MM一线治疗方案中逐步普及，患者的生存得以显著提高，但疾病仍不可治愈，各种新药在RRMM患者中不断涌现[8]。然而RCT和RWS数据存在不同程度的差异，疗效肯定、使用方便、不良反应易于管理的方案在RCT与RWS之间的差异较小，适用人群更为广泛。伊沙佐米自2018年在我国上市以来已在MM患者中得到广泛应用，本研究单中心数据显示，含伊沙佐米方案的疗效和安全性与RWS接近[8]–[9]，接受IRd方案治疗患者的PFS甚至优于伊沙佐米上市前中国延展研究（CCS）（8个月对6.7个月）[4]，且真实诊疗中更多高危细胞遗传学患者选择IRd方案。

多项来自欧洲的伊沙佐米RWS显示，IRd方案治疗RRMM的ORR为73％～74％，中位PFS时间为17.5～27.6个月，与TOURMALINE-MM1 RCT的疗效相当，疗效的差异主要源于患者基线情况及前线治疗不同[9]–[12]。与伊沙佐米在中国RRMM人群中的TOURMALINE-MM1 CCS相比，本研究纳入了较多75岁以上超高龄患者，CCS中ISS Ⅱ～Ⅲ期患者占63％，本研究患者比例高达80％；CCS中2～3线应用IRd方案患者占56％，本研究中比例为92.5％；CCS中难治病例占53％，本研究比例为67.4％；虽然本研究ORR低于CCS（40％对56％），但中位PFS时间优于CCS（8个月对6.7个月），中位OS时间优于CCS（未达到对25.8个月）[4]。本研究的总体疗效与杨扬等[6]报告的国内多中心RWS总体疗效相当。即使IRd方案治疗后患者病情进展，临床上仍可及时更换方案，通过伊沙佐米联合其他作用机制的药物（如达雷妥尤单抗或美法仑、泊马度胺等）提高治疗有效率，改善患者的生存[13]–[17]。

不同治疗线数亚组分析显示，二、三、四线及以上治疗的中位PFS时间分别为13、19、4个月，并非二线治疗PFS时间最长。考虑原因主要是11例高危CA患者中有8例在2线组，PFS时间仅4个月，这些患者均为一线VRd方案耐药、临床侵袭性复发，更换为含伊沙佐米方案不足以克服CA高危对疾病进程的影响，导致二线治疗组PFS差于三线治疗组。而三线治疗组患者中，CA标危患者比例达91％，持续治疗达6个疗程及以上的患者中位PFS时间达28个月；生化复发患者占69％，其中6例在持续治疗中缓解程度进一步加深。有4例患者虽然最佳疗效仅为SD，但坚持应用伊沙佐米方案治疗，疾病未进展，为后线治疗赢得了时间。

在不同治疗线数应用IRd方案患者的PFS与整体趋势一致，二、三、四线及以上治疗的中位PFS时间分别为8、14、3个月，因例数有限，亚组之间的差异无统计学意义。7例高危患者在VRD方案一线治疗进展后选择IRd方案作为二线方案，是二线治疗组疗效和生存率低于三线治疗组的主要原因。多因素分析表明，IRd方案治疗6个月内进展及前线治疗为来那度胺耐药的RRMM患者PFS时间短，与日本及国内多中心RWS结论一致[4],[18]，提示在临床实践中选择惰性复发、CA标危、非来那度胺耐药的RRMM患者进行IRd方案治疗更能获益。高龄、合并症多、肾功能不全、ECOG评分不影响IRd方案的疗效。

本研究显示伊沙佐米的PN发生率低，优于硼替佐米。3～4级AE发生率为19.2％，较CCS结果略低[4]，3例因严重腹泻继发感染停药，用药过程中出现的腹泻可能与药物累积剂量有关，2级或以上的腹泻建议应用蒙脱石散。IRd方案更易出现中性粒细胞、血小板降低等血液学不良反应，应及时予支持治疗。本研究患者均预防性予阿昔洛韦，带状疱疹发生率5.8％，MM1研究最初未要求应用阿昔洛韦，因带状疱疹发生率高进行方案修订[3],[13]。

本研究不足之处为单中心回顾性分析，样本量较少，治疗方案多样。尽管存在一些偏倚，但也反映了真实诊疗中医师的选择意向。高危或前线耐药患者首次复发时应用伊沙佐米方案难以获得满意效果，而惰性复发、标危、前线治疗敏感患者更能从包含伊沙佐米的三药方案中获益，且口服方案方便易行、对生活质量影响小，RWS数据接近甚至优于RCT，期待积累更多数据，规范MM的持续治疗策略。
